# Patient-specific 3D cryo(bio)printing of a glenoid labrum scaffold for fibrocartilaginous tissue engineering

**DOI:** 10.3389/fbioe.2026.1758582

**Published:** 2026-02-05

**Authors:** Francklin Trindade da Silva, Caio Moreira de Souza, Thiago Domingues Stocco

**Affiliations:** 1 Department of Internal Medicine, Faculty of Medical Sciences, State University of Campinas, Campinas, Brazil; 2 BioRegenera.Lab, Bioengineering Program, Scientific and Technological Institute, Brazil University, São Paulo, Brazil

**Keywords:** bioprinting, glenoid labrum, patient-specific modeling, shoulder injuries, tissue engineering

## Abstract

The glenoid labrum is a fibrocartilaginous structure essential for shoulder stability, yet its regeneration remains an unmet clinical challenge. Current surgical approaches restore initial joint stability but frequently fail to reestablish native biomechanics, leading to recurrence and early degenerative changes. In this study, we investigated the feasibility of fabricating a patient-specific, anatomically scaled glenoid labrum scaffold using digital modeling based on magnetic resonance imaging and 3D cryo(bio)printing of a gelatin methacryloyl (GelMA) hydrogel. Printing was performed in a temperature-controlled platform (22.5 °C, 15 °C, and −20 °C) to evaluate the influence of thermal conditions on structural fidelity and biological performance. Quantitative analyses showed that cryogenic deposition markedly improved printing precision, reducing filament spreading and enhancing geometric accuracy in both sharp-angle and grid-pattern evaluations. Biological assays indicated high viability of human mesenchymal stem cells under all temperature conditions, validating the cytocompatibility of the methodology. Morphological assessment by structured-light 3D scanning demonstrated that bioprinted patient-specific scaffold at −20 °C achieved the highest correspondence to the digital reference model. Overall, the integration of anatomical modeling with cryo(bio)printing proved to be an effective approach for producing anatomically faithful, patient-tailored scaffolds. This study presents the first demonstration of human glenoid labrum bioprinting and establishes a foundation for future translational research in fibrocartilaginous tissue regeneration.

## 1 Introduction

The glenoid labrum is a fibrocartilaginous ring that surrounds the glenoid cavity, deepening it and increasing the effective concavity by approximately 30%–50%, thereby expanding the contact surface and enhancing glenohumeral stability (Cooper et al., 1992). It exhibits considerable morphological variability and, histologically, demonstrates a regionally heterogeneous, gradually stratified fibrocartilaginous architecture rather than sharply demarcated layers, with an outer multidirectional superficial zone, a looser intermediate zone, and a denser collagenous core in which circumferentially oriented fibers provide tensile strength and contribute to reducing articular friction (Hill et al., 2008; Anthony et al., 2017; Hoang et al., 2023).

The mechanical properties of the glenoid labrum are crucial for its stabilizing function, as its structural integrity contributes significantly to joint containment forces. Even modest reductions in this contribution can alter shoulder biomechanics, increasing the reliance of the rotator cuff on compressive stabilization mechanisms (Halder et al., 2001).

Glenoid labrum injuries are common, arising from acute trauma such as dislocations or from repetitive microtrauma associated with overhead movements. Among these, superior labrum anterior-to-posterior (SLAP) lesions are frequent in throwing athletes, while Bankart lesions typically accompany anterior shoulder dislocations, with an incidence ranging from 87% to 100% in first-time cases (Michener et al., 2018; Hu et al., 2023). Clinically, patients often present with diffuse pain, instability, restricted range of motion, and mechanical symptoms such as clicking or locking. Particularly, the chronic instability alters load distribution across the joint, promoting cartilage degeneration and ultimately leading to glenohumeral osteoarthritis, a highly disabling condition (Sofu, 2014; Cho et al., 2017).

The current standard treatment for glenoid labrum injuries is surgical repair using suture anchors, applied to both Bankart and SLAP lesions to restore initial joint stability. However, recurrence rates of shoulder instability range from 10% to 20%, and there is a high incidence of early degenerative changes, such as post-instability arthropathy (Co et al., 2023; Victor et al., 2025). Surgical complications may include neurological injury, anchor loosening, and foreign-body–induced synovitis, all of which can lead to chondrolysis (Matsuki and Sugaya, 2015). These outcomes highlight the limitations of current procedures in fully restoring the native biomechanics and biology of the labrum, reinforcing the need for innovative regenerative approaches.

Tissue engineering has emerged as a promising strategy for glenoid labrum regeneration by combining cells, biomaterials, and biochemical cues within biodegradable three-dimensional scaffolds (Nordberg et al., 2024). Such scaffolds, typically composed of hydrogels, polymers, or ceramics, provide both structural support and biological stimulation. For labral repair, it is essential that these constructs replicate not only the native biological features but also the morphological architecture of the tissue (Caruso et al., 2025). This has become increasingly feasible with recent advances in biofabrication technologies that enable the design of patient-specific scaffolds based on medical imaging, thereby accommodating anatomical variability and promoting functional integration.

In this context, three-dimensional (3D) bioprinting has emerged as a transformative biofabrication technology capable of precisely depositing biomaterials and, when applicable, living cells in a layer-by-layer manner to generate constructs with highly controlled architectures. Guided by digital models derived from medical imaging, this technique enables the creation of patient-specific geometries that accurately reproduce the morphological and structural features of native tissues. By allowing spatial control of material distribution and internal gradients, 3D bioprinting provides a powerful platform for fabricating anatomically faithful scaffolds with tunable mechanical and biological properties, advancing the field of regenerative medicine (Zhang et al., 2021; Loukelis et al., 2024).

Recent advances have demonstrated applications of 3D bioprinting in the regeneration of fibrocartilaginous tissues such as the meniscus (Stocco et al., 2022) and intervertebral disc (Moxon et al., 2025). However, to our knowledge, no previous study has investigated the use of 3D bioprinting specifically for the regeneration of the glenoid labrum. Thus, the present work therefore aims to develop, for the first time, a patient-specific bioartificial glenoid labrum using digital anatomical modeling based on medical imaging and bioprinting methodology, addressing a critical unmet need in shoulder tissue engineering.

To achieve this, we employed 3D cryo(bio)printing, an advanced approach that utilizes low-temperature deposition to induce immediate solidification of printed layers (Luo et al., 2022; 2024). We hypothesize that printing at low temperatures, particularly at −20 °C, enhances scaffold shape fidelity by promoting rapid stabilization of the printed structure and minimizing deformation during fabrication, while not compromising cellular viability when combined with an appropriate cryoprotective formulation. By integrating patient-specific anatomical modeling with cryo(bio)printing, this work establishes a reproducible strategy for fabricating anatomically accurate glenoid labrum scaffolds, providing a technological foundation for future regenerative applications aimed at restoring labral function and joint stability.

## 2 Materials and methods

**FIGURE 1:**
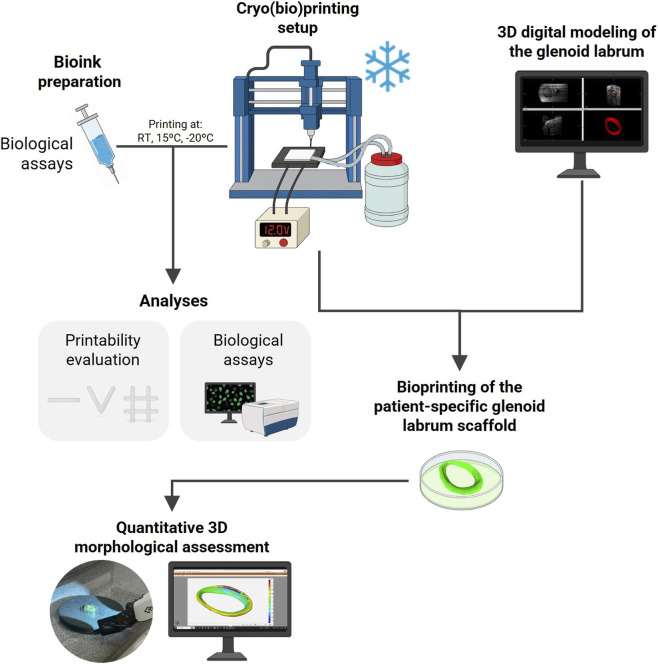
Schematic overview of the experimental workflow for patient-specific cryo(bio)printing of the glenoid labrum. The process involved digital reconstruction of the labrum from magnetic resonance imaging data, preparation of a hydrogel-based bioink, 3D cryo(bio)printing under controlled temperature conditions, and subsequent assessments of printability, cell viability, and quantitative 3D morphological fidelity.

The overall methodology employed in this study is summarized schematically in Figure 1. Each stage of the process is described in detail in the following subsections.

### 2.1 Cryo(bio)printing system setup

The cryo(bio)printing system used in this study was adapted from a previously described design (Luo et al., 2024) and assembled on a commercial 3D bioprinter (RevX, BioEdTech, Brazil). The setup consisted of a thermoelectric Peltier plate connected to an adjustable power supply that allowed precise control of the cooling intensity. The plate was mounted on a base equipped with a chilled-water circulation system driven by a submersible pump, with ice added to enhance thermal dissipation efficiency. The surface temperature of the printing platform was monitored using both a non-contact infrared thermometer and a contact thermometer to determine and standardize the optimal printing conditions.

### 2.2 Preparation of the hydrogel-based ink

Gelatin methacryloyl (GelMA) was selected as the biomaterial matrix for ink formulation due to its favorable properties and extensive use in biofabrication studies (Das et al., 2024). GelMA was synthesized following the general procedure established in our previous studies (Montesdeoca et al., 2020; de Silva et al., 2024; da Silva et al., 2025). Briefly, type A gelatin derived from porcine skin (Sigma-Aldrich, United States) was dissolved in phosphate-buffered saline (PBS; Sigma-Aldrich, United States) at 50 °C under continuous stirring until a uniform solution was obtained. Methacrylic anhydride (Sigma-Aldrich, United States) was then added dropwise to the mixture and allowed to react for approximately 3 h at 50 °C under constant agitation. The reaction mixture was subsequently diluted with preheated PBS, followed by dialysis against deionized water at 40 °C for 4 days, using 12–14 kDa molecular weight cut-off (MWCO) membranes (Spectra/Por™ 2 RC Dialysis Membrane Tubing, Fisher Scientific, United States), with the water replaced daily. The dialyzed solution was finally frozen and lyophilized, yielding GelMA as a dry, white porous solid suitable for storage.

For the preparation of the printable hydrogel, GelMA was reconstituted in PBS at a final concentration of 80 mg mL^-1^, and Lithium phenyl-2,4,6-trimethylbenzoylphosphinate (LAP; Sigma-Aldrich, United States) was incorporated as a photoinitiator at a concentration of 5 mg mL^-1^ to enable subsequent photocrosslinking during the bioprinting process.

### 2.3 Temperature-controlled bioprinting

**FIGURE 2:**
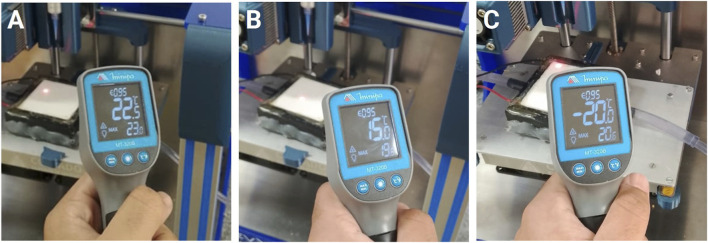
Surface temperature profiles of the printing platforms under different experimental conditions: **(A)** room temperature (RT, 22.5 °C), **(B)** refrigerated platform (15 °C), and **(C)** frozen platform (−20 °C). Each condition represents the thermal environment applied during temperature-controlled bioprinting.

To evaluate the influence of substrate temperature, bioprinting was performed on platforms maintained at three distinct thermal conditions: room temperature (RT, 22.5 °C), refrigerated (15 °C), and frozen (−20 °C). These temperature settings were chosen based on several previous studies that reported GelMA bioprinting under comparable thermal environments (Ding et al., 2019; Janmaleki et al., 2021; Luo et al., 2024). The corresponding thermal profiles of the printing platforms are illustrated in Figure 2.

Digital models were processed using PrusaSlicer software version 2.6.1 (Prusa Research, Czech Republic) to generate the G-code files for printing. The bioprinting process was performed using a nozzle with an internal diameter of 0.4 mm, a layer height of 0.4 mm, and a printing speed of 8 mm/s. The printhead operated at room temperature under all conditions.

*In situ* photocrosslinking of the printed hydrogel was carried out by exposure to light with a wavelength of 405 nm immediately after layer deposition. Each deposited layer was briefly irradiated for approximately 5–10 s to induce partial crosslinking and prevent filament diffusion. Upon completion of the entire scaffold, the construct was exposed to a total light dose of approximately 60 s to ensure complete crosslinking and structural stabilization.

#### 2.3.1 Printability evaluation

**FIGURE 3:**
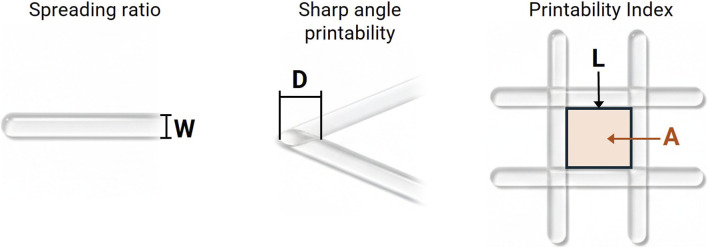
Schematic representation of the printability parameters evaluated in this study. Spreading Ratio: quantifies the lateral expansion of the extruded filament (W) relative to the nozzle diameter. Sharp Angle Printability: measures the ability of the printed material to preserve the designed 30° intersection, with D representing the measured distance between filament edges. Printability Index: assesses the geometric fidelity of grid-patterned constructs, where L and A correspond to the perimeter and area of each pore, respectively.

Printability was evaluated through three complementary analyses designed to assess the dimensional accuracy and geometric fidelity of the printed structures: the Spreading Ratio, the Angle Printability, and the Printability Index (He et al., 2016; Habib et al., 2018; Naghieh et al., 2020; Bom et al., 2022). These parameters are illustrated in Figure 3 and described in detail below.

##### 2.3.1.1 Spreading ratio

The Spreading Ratio quantifies the tendency of the extruded filament to undergo lateral expansion relative to the nozzle diameter. It is defined as the ratio between the actual width of the printed filament ( Wprinted ) and the internal diameter of the extrusion nozzle ( Wnozzle ), as expressed in the following equation:

$$Spreading\;ratio=\frac{Wprinted}{Wnozzle}$$

Values greater than 1.0 indicate that the material spread after deposition, producing filaments wider than the nozzle opening, whereas a value equal to 1.0 represents the ideal condition, reflecting the absence of lateral spreading. Measurements of the filament width were obtained from printed line patterns (Figure 3), with three random regions analyzed along each line, and the mean value recorded for subsequent analysis.

##### 2.3.1.2 Sharp angle printability

The Sharp Angle Printability assesses the ability of the printed material to preserve the designed angular geometry after deposition. Standardized patterns containing 30° internal angles were printed, and the corresponding measurement region (D) was defined as the shortest distance between the inner edges of the filaments forming the intersection, as illustrated in Figure 3. The resulting structures were imaged under controlled lighting conditions for dimensional analysis. This parameter is defined as the ratio between the measured distance at the printed intersection ( Dprinted ) and the corresponding distance in the digital model ( Dmodel ), as expressed below:

$$Sharpe\;Angle\;Printability=\frac{Dprinted}{Dmodel}$$

Values close to 1.0 indicate high shape fidelity and structural stability, whereas deviations reflect geometric distortion caused by filament spreading or insufficient hydrogel solidification.

##### 2.3.1.3 Printability index

The Printability Index evaluates the geometric fidelity of printed two-dimensional grid patterns composed of orthogonal filaments (0°/90°) (Figure 3). It is defined based on the morphometry of the pores formed by the intersection of the filaments and calculated according to the following equation, where L represents the perimeter and A the average area of the pores:

$$Printability\;index=\frac{L^{2}}{16A}$$

A value equal to 1.0 represents the ideal printing condition, corresponding to perfectly square pores as designed in the digital model. Values below 1.0 indicate structural collapse, typically resulting from filament fusion that produces smaller or rounded pores, whereas values above 1.0 suggest geometric distortion associated with excessively thin filaments or deposition defects that create abnormally large pores. This index therefore serves as a quantitative indicator of the material’s ability to maintain regular two-dimensional printing patterns without distortion caused by spreading or flow instabilities. Measurements of the Printability Index were performed on printed grid structures consisting of four parallel filaments in each direction (5 × 5). All pores within each sample were analyzed to determine the mean value for each experimental condition.

Image acquisition was performed under standardized lighting and magnification, and quantitative measurements were obtained using the ImageJ/Fiji software (National Institutes of Health, United States) after spatial calibration with a reference scale. For visual contrast and group identification during imaging, water-based dyes were added to the hydrogel inks, assigning green to 22.5 °C, orange to 15 °C, and pink to −20 °C.

#### 2.3.2 Biological assays

##### 2.3.2.1 Cell culture

Human mesenchymal stem cells (hMSCs; Merck, United States) were cultured in Dulbecco’s Modified Eagle Medium (DMEM; Merck, United States) supplemented with 10% (v/v) fetal bovine serum (FBS; Merck, United States), 1 mM L-glutamine, and 1% antibiotic–antimycotic solution (Gibco, United States). The cells were maintained under standard culture conditions in a humidified incubator at 37 °C with 5% CO_2_. The medium was replaced every 2 days, and cells were subcultured upon reaching approximately 80% confluence using a 0.25% trypsin–EDTA solution (Merck, United States). Cell expansion was performed up to the fourth passage (P4) to ensure phenotypic stability for subsequent bioink preparation.

##### 2.3.2.2 Bioink preparation

The hydrogel solution described in Section 2.2 was used as the base material for bioink formulation. To provide cryoprotection during the cryo(bio)printing process, 10% (v/v) dimethyl sulfoxide (DMSO) and 8% (w/v) D-(+)-melezitose hydrate were incorporated into the prepolymer solution (Luo et al., 2022; Luo et al., 2024; Weygant et al., 2024). The hMSCs were then suspended in the hydrogel solution at a final density of 1 × 10^6^ cells. mL^-1^ under sterile conditions and subsequently loaded into 5 mL sterile syringes compatible with the bioprinter for printing procedures.

##### 2.3.2.3 Cell viability

For the cell viability assessment, hMSC-laden scaffolds were bioprinted using the same printing parameters described in Section 2.3 and the same grid pattern employed for the Printability Index evaluation. After printing, the constructs were incubated in standard culture medium for 1 and 7 days at 37 °C in a humidified atmosphere containing 5% CO_2_.

Cell viability was evaluated using the Live/Dead Viability/Cytotoxicity Kit (Thermo Fisher Scientific, United States) according to the manufacturer’s instructions. At each time point, scaffolds were gently rinsed with PBS and incubated for 30 min at 37 °C in the dark with calcein-AM (0.5 μg mL^-1^) and ethidium homodimer-1 (EthD-1, 2.0 μg mL^-1^). After staining, samples were washed again with PBS, and fluorescence images were acquired immediately using an inverted fluorescence microscope (Eclipse Ti, Nikon, Japan). Image analysis was performed using ImageJ software (NIH, United States), and cell viability was quantified as the percentage of live (green) cells relative to the total cell population (green + red).

### 2.4 3D digital modeling of the glenoid labrum

As the primary goal of this study was to bioprint a patient-specific glenoid labrum, a three-dimensional digital model of the labral structure was first generated from Magnetic Resonance Imaging (MRI) scan of the right shoulder obtained from an anonymized database. The Digital Imaging and Communications in Medicine (DICOM) files were imported into *InVesalius three software* (CTI/MCTI, Brazil), an open-source software for medical image visualization and 3D reconstruction. Semi-automatic segmentation was performed to isolate the glenoid labrum from surrounding tissues, followed by manual refinement to ensure anatomical accuracy.

The segmented labrum was then reconstructed as a three-dimensional volumetric model and converted into a surface mesh. The final model was exported in STL (Standard Triangle Language) format, representing the anatomical geometry of the glenoid labrum to be used as the digital basis for subsequent biofabrication planning.

### 2.5 Bioprinting of the patient-specific glenoid labrum scaffold

The patient-specific glenoid labrum scaffold was bioprinted using the previously generated 3D digital model, the same bioink formulation and the printing parameters detailed in the previous sections. The bioprinting process was carried out on platforms maintained at three temperature conditions (22.5 °C, 15 °C, and −20 °C), as described earlier.

### 2.6 Quantitative 3D morphological assessment

To assess the morphological accuracy of the printed scaffold in reproducing the patient-specific geometry of the glenoid labrum, a quantitative comparison was performed between the original 3D digital model and the corresponding printed construct. The labrum scaffolds were scanned using a structured-light 3D scanner (Dolphin, RVS3D, Brazil) with a scanning accuracy of 0.04 mm and a point spacing of 0.06 mm. Each specimen was scanned in three orientations and twelve rotational positions on a motorized turntable to ensure complete surface acquisition.

The resulting digital reconstructions were exported in STL format and imported into GOM Inspect software (GOM GmbH, Germany) for metrological analysis. After fitting and alignment with the nominal 3D model derived from MRI data, a 3D deviation analysis was performed using the CAD Comparison tool. The deviation maps were visualized as color-coded chromatograms, allowing quantitative evaluation of the morphological fidelity between the printed construct and the original digital geometry.

### 2.7 Statistical analysis

All experiments, including printability, biological, and morphological analyses, were performed in triplicate. Data are presented as mean ± standard deviation. Statistical comparisons among groups corresponding to different printing temperatures (22.5 °C, 15 °C, and −20 °C) were performed using one-way analysis of variance (ANOVA) followed by Tukey’s *post hoc* test. Statistical significance was considered at *p* < 0.05. All analyses and graphical representations were performed using GraphPad Prism software (version X; GraphPad Software, United States).

## 3 Results and discussion

The first step of this study was to investigate the feasibility of 3D cryo(bio)printing as a biofabrication strategy capable of reproducing anatomically complex structures with high fidelity, such as the glenoid labrum. Quantitative morphological analyses were performed to evaluate the printing performance under different temperature conditions, aiming to determine whether temperature control during the cryo(bio)printing process could enhance the geometric stability and dimensional precision of the printed constructs.

**FIGURE 4:**
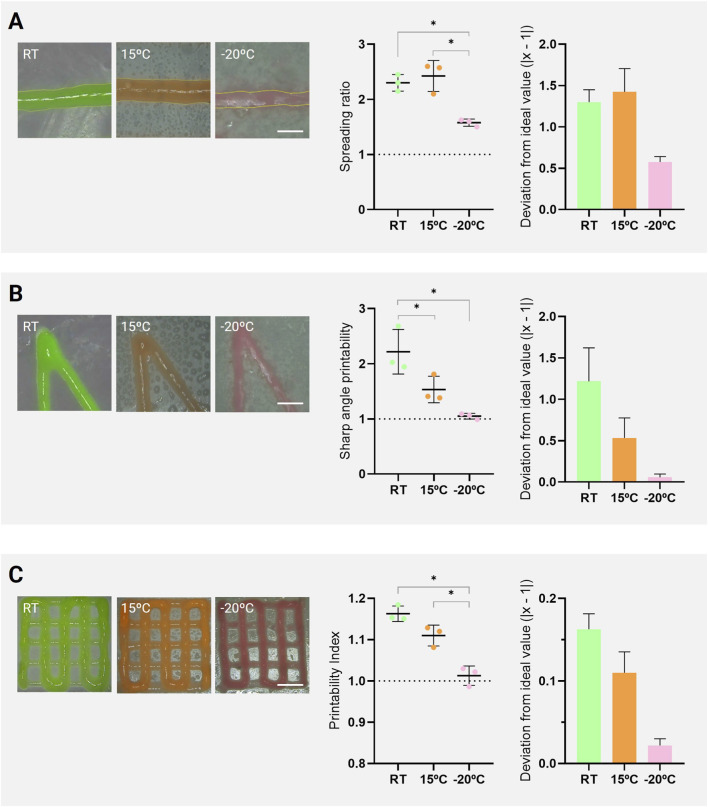
Printability analyses of GelMA constructs bioprinted at different substrate temperatures: room temperature (RT, 22.5 °C), 15 °C, and −20 °C. **(A)** Spreading ratio, **(B)** sharp angle printability, and **(C)** printability index. Representative images (left) show the printed geometries at each condition (scale bars: A = 1 mm, B = 4 mm, C = 6 mm). The corresponding graphs (middle) present the quantitative values for each parameter, where dashed lines indicate the theoretical ideal value (1.0) and asterisks denote statistically significant differences between groups (*p < 0.05). Additionally, bar graphs (right) display the absolute deviation from the ideal value (1.0) to facilitate visualization and interpretation of the data.

The analysis of the Spreading Ratio (Figure 4A) revealed significant differences among the tested temperature conditions. Cryo(bio)printing at −20 °C yielded a mean value closer to the ideal (1.58 ± 0.07), reflecting reduced lateral expansion of the extruded filament and greater control over bioink deposition. In contrast, printing at 15 °C (2.43 ± 0.28) and at room temperature (2.30 ± 0.15) resulted in greater spreading and lower geometric stability. This trend indicates that immediate solidification on the frozen platform acts as a physical constraint on hydrogel diffusion, limiting molecular mobility and consequently reducing filament expansion. Consistent results have been reported by other authors in previous studies. Santana et al. (Santana et al., 2024) observed that rapid freezing of GelMA-based bioinks during cryo(bio)printing improved the structural integrity of printed constructs, even in formulations modified with graphene, which could potentially alter extrusion dynamics. Similarly, Weygant et al. (Weygant et al., 2024), although employing a different bioprinting approach (droplet-based bioprinting), demonstrated that droplet deposition onto a cryogenic platform produced instantaneous freezing, preventing droplet coalescence and enhancing lateral resolution in printed structures. Overall, these results demonstrate that temperature regulation directly influences filament morphology during extrusion, with cryogenic conditions providing superior control over material deposition.

The evaluation of geometric fidelity through the Sharp Angle Printability (Figure 4B) also demonstrated the superior performance of cryo(bio)printing in preserving acute angles. Constructs printed at −20 °C showed a mean value of 1.05 ± 0.05, whereas those fabricated at RT and 15 °C exhibited average values of 2.22 ± 0.40 and 1.53 ± 0.24, respectively. Although no statistically significant difference was observed between the −20 °C and 15 °C groups, the cryogenic condition still exhibited a trend toward improved angular definition, likely associated with the immediate solidification induced by the cooled platform, which stabilizes the material upon deposition and minimizes structural deformation before photocrosslinking. Previous studies have emphasized the importance of angular geometry as a critical indicator of bioprinting accuracy. Naghieh et al. (2020) systematically analyzed angular patterns ranging from 25° to 90°, demonstrating that acute geometries are particularly sensitive to temperature and bioink properties, often showing reduced definition when thermal regulation is absent. Similarly, Malekpour and Chen, (2022) reported that overlap and structural collapse in sharp corners are mitigated when faster gelation occurs immediately after extrusion, improving the accuracy of internal angles. Under this perspective, the results presented here indicate that cryo(bio)printing enhances the preservation of edge and vertex definition in low-temperature conditions, supporting its potential use in applications that require high anatomical precision, such as patient-specific reconstruction of the glenoid labrum.

The analysis of grid-patterned constructs (Figure 4C) showed that the Printability Index approached the ideal value (1.0) as the printing temperature decreased: RT = 1.16 ± 0.02, 15 °C = 1.08 ± 0.05, and −20 °C = 1.02 ± 0.04, with statistically significant differences between the −20 °C group and the other conditions. In practical terms, the cryogenic condition reduced filament width variability and better preserved pore openness, resulting in more uniform grids with higher geometric fidelity. Pore size and area analyses in printed grids have been widely used as indicators of shape fidelity, demonstrating that both material formulation and processing adjustments can minimize deviations from the original CAD design (40, 41). These findings reinforce the importance of temperature control as a key variable in maintaining dimensional fidelity, particularly in hydrogel-based bioinks whose rheological behavior is strongly influenced by temperature and gelation kinetics.

Despite the consistent improvement observed across all printability metrics at −20 °C, none of the evaluated parameters fully reached their theoretical ideal values. This indicates that while cryo(bio)printing substantially enhances geometric control, residual deviations from the designed geometry persist even under cryogenic conditions. Such limitations are likely associated with the intrinsic rheological properties of GelMA at the selected concentration and the absence of active cooling at the nozzle tip. Therefore, the results highlight cryo(bio)printing as an effective strategy to improve shape fidelity, while also emphasizing the need for further optimization of material formulation, and thermal gradients to approach ideal geometric accuracy.

**FIGURE 5:**
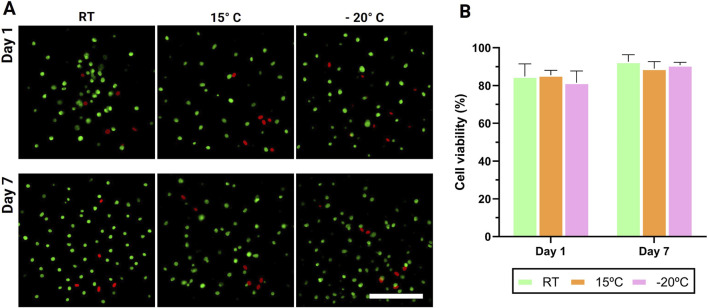
Live/Dead fluorescence analysis of human mesenchymal stem cells encapsulated in GelMA scaffolds bioprinted under different temperature conditions: room temperature (RT, 22.5 °C), 15 °C, and −20 °C. **(A)** Representative fluorescence micrographs show viable cells (green) and non-viable cells (red) after 1 and 7 days of culture. **(B)** The quantitative graph (right) presents the mean cell viability (%) for each condition, demonstrating no significant differences among groups at either time point (p > 0.05). Scale bar = 450 μm.

Live/Dead fluorescence imaging at 1 and 7 days showed high viability across all temperature conditions (Figure 5). The proportion of live cells ranged from 82% to 85% at 24 h and exceeded 90% by day 7, indicating preservation and apparent recovery of hMSCs after printing. No statistically significant differences were detected among the room temperature, 15 °C, and −20 °C groups at either time point (*p* > 0.05), demonstrating that the temperature settings used during printing did not compromise cytocompatibility. These findings are consistent with previous cryo(bio)printing reports that achieved high post-printing viability for hMSCs and other cell types under low-temperature deposition and rapid solidification regimes (Luo et al., 2022; 2024; Ravanbakhsh et al., 2022). Those studies employed the same cryoprotective formulation based on DMSO and Melezitose, which was also used in the present work. Together, the results support the potential feasibility of temperature-controlled 3D cryo(bio)printing for producing cell-laden GelMA constructs without detrimental effects on short-term cell survival.

It should be noted that the biological evaluation in this study was intentionally limited to short-term cell survival assessed by Live/Dead staining at early culture time points. This choice reflects the primary objective of the biological assays, which was to verify the acute cytocompatibility of the cryo(bio)printing process and the applied temperature conditions, rather than to assess long-term cell behavior or tissue maturation. Early post-printing viability is a critical parameter in cryo(bio)printing, as it directly reflects the cellular response to thermal stress, cryoprotective agents, and rapid solidification during deposition. Accordingly, no conclusions are drawn regarding cell proliferation, lineage-specific differentiation, extracellular matrix production, or functional tissue regeneration, which will require dedicated long-term and functional studies beyond the scope of the present work.

**FIGURE 6:**
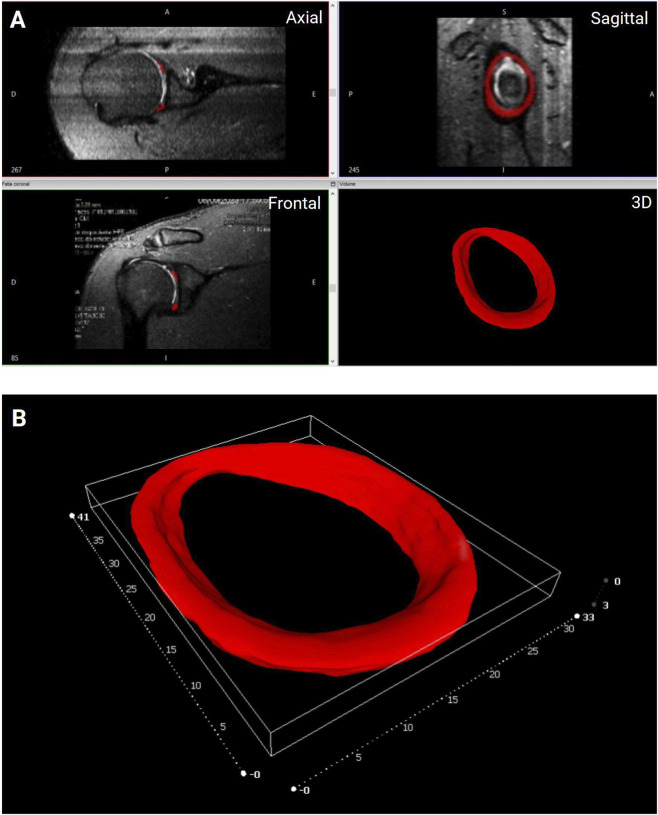
Generation of the 3D digital model of the glenoid labrum based on magnetic resonance imaging data. **(A)** Segmentation of the labrum in InVesalius software, showing the anatomical isolation in axial, sagittal, and frontal planes, and the corresponding 3D reconstruction. **(B)** Final volumetric rendering of the 3D model, representing the patient-specific geometry of the glenoid labrum used for subsequent printing steps. Scale in millimeters.

The 3D reconstruction of the glenoid labrum was performed using MRI data from a human shoulder and processed in InVesalius software (CTI Renato Archer, Brazil), as illustrated in Figure 6. This open-source platform has been widely adopted in both clinical and experimental settings for anatomical segmentation and 3D reconstruction, being recognized for its intuitive interface and satisfactory accuracy, particularly for structures with high morphological complexity such as the skull, spine, and joints (Buffinton et al., 2023; Yap Abdullah et al., 2024). Although other established tools, including Amira, 3D Slicer, and ITK-SNAP, are also commonly employed for this purpose, InVesalius has shown, in our previous experience, performance compatible with the geometric precision required for patient-specific biofabrication of musculoskeletal models (Stocco et al., 2022; da Silva et al., 2025).

During the segmentation process, the automatic thresholding feature of the software served only as an initial step for defining the glenoid labrum boundaries. Extensive manual refinements were required to adjust the contours and ensure accurate anatomical representation. Such fidelity is particularly critical for the biofabrication of patient-specific scaffolds, as even small geometric inaccuracies may compromise the functional performance of the printed construct. Similar challenges have been reported in previous studies involving the segmentation of complex joint structures, especially within the shoulder, where the low contrast of fibrocartilaginous tissues in MRI scans necessitates manual intervention to preserve morphological accuracy (Dowe et al., 2024; da Silva et al., 2025). Moreover, differentiating the superior insertion of the long head of the biceps tendon, closely associated with the superior portion of the labrum, requires detailed anatomical knowledge to avoid misinterpretation, a relationship extensively discussed in prior anatomical investigations (Diplock et al., 2023).

The dimensional parameters of the 3D digital model of the glenoid labrum are summarized in Table 1 and compared with the anatomical ranges reported in previous studies (Frankle et al., 2009; Anthony et al., 2017; Koga et al., 2020). Overall, the dimensions obtained from the reconstructed model were consistent with the anatomical intervals described in cadaveric and MRI-based analyses. The model accurately reproduced the characteristic circular morphology of the labrum, with an outward projection that extends the glenoid rim and increases the effective articular diameter, in agreement with previous anatomical descriptions. These results confirm the anatomical adequacy of the reconstructed model and support its suitability for subsequent stages of patient-specific bioprinting.

**TABLE 1:** Dimensional comparison between the 3D digital model of the glenoid labrum obtained in this study and the anatomical ranges reported in the literature.

Measurement	Model (mm)	Reported anatomical range (mm) Frankle et al. (2009), Anthony et al. (2017), Koga et al. (2020)
Mediolateral width(x-axis)	33.0	29.0–45.0
Anteroposterior length(y-axis)	41.0	31.0–45.0
Superoinferior thickness (Z-axis)	3.0	3.0–7.0

**FIGURE 7:**
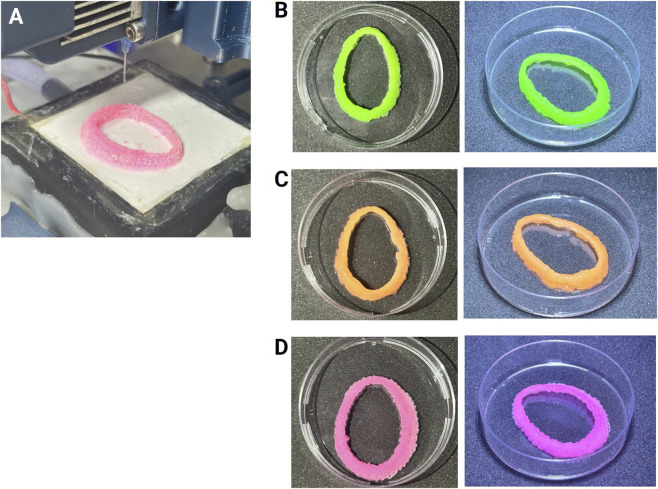
**(A)** Macroscopic view of the printing process and the resulting patient-specific glenoid labrum scaffolds produced under different temperature conditions. **(B–D)** Representative constructs bioprinted at room temperature (22.5 °C, green), 15 °C (orange), and −20 °C (pink), respectively. Scaffolds are shown in 60 mm Petri dishes for scale reference.

Figure 7 shows the macroscopic appearance of the bioprinted constructs produced under the three temperature conditions. In all groups, the overall circular geometry and external wall continuity were satisfactorily preserved, indicating that the global configuration of the labrum models could be maintained regardless of the temperature employed. This observation suggests that for global geometries and regions of low topographical complexity, the bioprinting process is relatively robust to moderate temperature variations.

**FIGURE 8:**
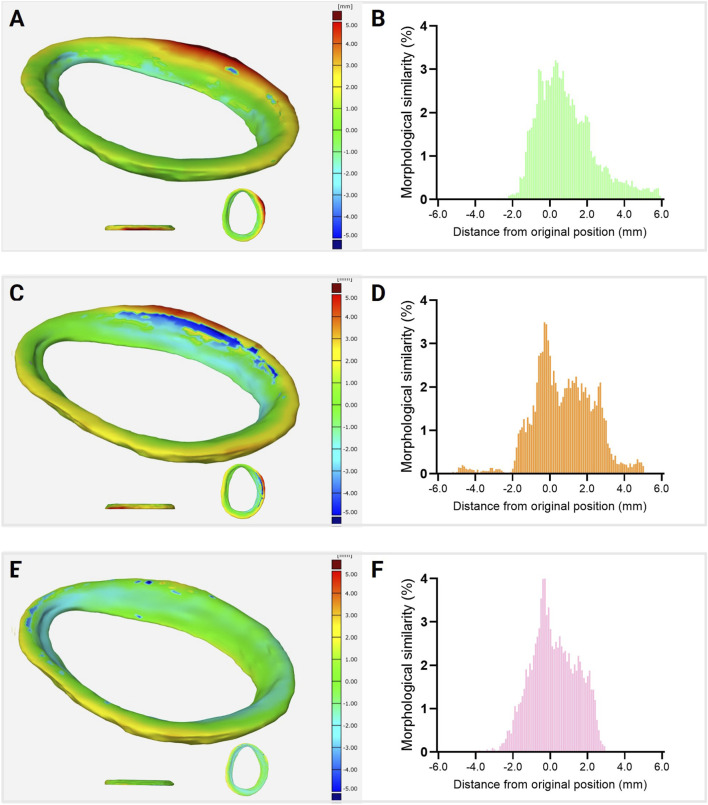
Quantitative 3D morphological comparison between the printed glenoid labrum scaffolds and the original digital model. Color maps represent spatial deviation distributions for scaffolds printed at **(A)** room temperature (22.5 °C), **(C)** 15 °C, and **(E)** −20 °C, showing local differences in surface geometry relative to the reference model (in millimeters). The corresponding histograms **(B,D,F)** display the statistical distribution of deviations for each condition, evidencing higher morphological accuracy at lower printing temperatures.

However, more pronounced differences were observed in the quantitative 3D morphological assessment. This analysis compared the bioprinted glenoid labrum scaffolds fabricated under different temperature conditions with the digital reference model derived from medical image segmentation. The color-coded deviation maps (Figures 8A,C,E) visually illustrate the spatial discrepancies between the printed and digital models, while the corresponding histograms (Figures 8B,D,F) provide a statistical representation of the deviation distribution.

Quantitative analysis of geometric fidelity revealed that cryo(bio)printing at −20 °C achieved the highest correspondence with the original digital model, with 90.79% of the surface points located within a ±2 mm deviation range. In comparison, scaffolds printed at 15 °C and room temperature showed 80.57% and 75.37% of points within the same range, respectively. These results demonstrate that controlled cooling of the printing platform enhances the preservation of three-dimensional morphology, particularly in regions with pronounced curvature or steep inclination. The ±2 mm deviation range was adopted as a comparative metric to assess global shape fidelity rather than as a definitive clinical tolerance. Given the thinness of the labrum, its complex circumferential geometry, and the inherent dimensional variability of hydrogel-based constructs, this threshold allows robust discrimination among printing conditions while minimizing overinterpretation of localized surface deviations. Collectively, the findings confirm that cryo(bio)printing is an effective strategy for accurately replicating anatomically complex structures such as the glenoid labrum, enabling superior morphological correspondence between the printed construct and the reference anatomy, which is a critical requirement in the biofabrication of fibrocartilaginous tissues with biomechanical relevance.

Although positive outcomes were achieved, some methodological and operational limitations should be acknowledged. The segmentation of the glenoid labrum required extensive manual refinement, as automatic thresholding algorithms were insufficient to accurately isolate the target structure. This highlights the need for more advanced tools capable of automating segmentation and model reconstruction, which may be addressed in future studies through artificial intelligence–based approaches, particularly through machine learning algorithms trained on anatomically labeled datasets (Diaz-Pinto et al., 2024). Maintaining precise thermal control of the printing platform also posed a technical challenge, requiring continuous calibration to ensure temperature stability during extrusion. In addition, cleaning the printing surface between samples was necessary to prevent adhesion artifacts, making the workflow more labor-intensive.

In the present study, the patient-specific workflow was demonstrated using a single MRI-derived glenoid labrum model. This choice reflects the individualized nature of patient-specific fabrication, in which the digital reconstruction and biofabrication pipeline is applied on a case-by-case basis rather than optimized for population-level generalization. While the use of a single dataset is sufficient to demonstrate the technical feasibility of the proposed workflow, it does not capture the full spectrum of anatomical variability observed across different patients. Therefore, broader validation using multiple MRI datasets, including variations in patient anatomy, demographics, and pathological conditions, will be required in future studies to assess the robustness and scalability of this approach.

Finally, advancing this research toward translational validation will require further steps beyond the scope of the present study, including optimization of scaffold mechanical properties to withstand physiological loading and long-term evaluation of cell behavior and differentiation. These future efforts will be essential to reinforce the clinical potential of this methodology for patient-specific regeneration of fibrocartilaginous tissues.

Despite these challenges, the benefits observed clearly outweigh the limitations, particularly when considering the translational potential of the technique and the pioneering nature of this work. Although previous studies have addressed tissue engineering strategies for glenoid labrum repair with encouraging outcomes (Wahab et al., 2019; Co et al., 2023; Caruso et al., 2025), this work represents, to our knowledge, the first investigation of the feasibility of human glenoid labrum bioprinting. The scaffolds were fabricated at real anatomical scale and designed in a patient-specific manner based on medical imaging data, employing 3D cryo(bio)printing as a key technique to ensure structural fidelity without compromising cell viability. This achievement represents a significant step toward the development of customized grafts for complex fibrocartilaginous structures, with clear potential for future translation into clinical practice.

## 4 Conclusion

This study demonstrates the feasibility of fabricating a patient-specific, anatomically scaled glenoid labrum scaffold by integrating MRI-based 3D modeling with temperature-controlled cryo(bio)printing of a GelMA hydrogel. Quantitative printability analyses showed that low-temperature deposition improved geometric fidelity, with reduced filament spreading, superior preservation of sharp angles, and grid patterns approaching the ideal morphology. The cryogenic condition also yielded the highest correspondence between printed constructs and the digital reference model, with most surface points falling within a defined deviation range. Importantly, high cell viability was maintained in short-term culture across all temperature conditions, confirming the acute cytocompatibility of the cryo(bio)printing process and bioink formulation. Collectively, these findings establish a robust proof of concept for producing anatomically faithful, patient-specific labral scaffolds and provide a technical basis for future investigations focused on mechanical optimization and extended biological validation prior to translational application.

## Data Availability

The raw data supporting the conclusions of this article will be made available by the authors, without undue reservation.
